# The Congress on Tuberculosis

**Published:** 1899-06-03

**Authors:** 


### The Hospital

A Journal of

The Medical Sciences and Hospital Administration.

Vol. XXVI.—No. 662.] Edited by Sir Henry Burdett, K.C.B., and Solomon C. Smith, M.D., M.R.C.P. [June 3, 1899.

### The Congress on Tuberculosis.

The meetings of the International Congress on the Prevention and Cure of Tuberculosis, which assembled last week at Berlin, are not likely to make any sensible addition to the sum of human knowledge on either of the two heads to which the proceedings are referred, inasmuch as such additions could only be derived either from the laboratory of the bacteriologist or from the practice of the physician. The chief value of the work of the Congress must depend upon the influence which it may exert upon opinion, both professional and public, and upon the degree in which it may systematise knowledge with a view to its more prompt and practical application. There are few more instructive chapters in medical history than that which contains an account of the changes of belief concerning tuberculosis, and especially concerning tuberculosis of the lungs, which have been brought about during the last half of this expiring century. Fifty years ago the prevailing doctrine in England was that consumption, as it was almost universally called, was absolutely non-contagious, and largely a result of inherited constitutional defect; and any precautions to prevent its extension from the diseased to the healthy would have been deemed alike futile and superfluous by the great majority of practitioners. The opposite view which prevailed in some Continental countries, in which the families of persons who died of "English disease," as it was often described, were mulcted in heavy expenses for the disinfection of rooms, and for the destruction of furniture and utensils which had come in contact with the sick, was commonly regarded as an example of gross and ignorant superstition, chiefly kept alive by the opportunities for extortion which it continually afforded. The genius of William Budd, indeed, had discerned at least an outline of the truth; and, in the days when bacilli were as yet not only unknown but unthought of, he proclaimed his conviction that consumption was not inherited, but that it was "a zymotic disease with a long period of incubation," and that it was eminently contagious; and he illustrated this position by the history of its diffusion when newly introduced into populations in no way fortified against it by acquired power of resistance to its virus. His wisdom cried aloud in the streets, but it is not too much to say that no man regarded her. In England, which once either was or was alleged to be the special home of the disease, it began to be realised that its prevalence could to a large extent be controlled by improved sanitary conditions, by drainage, by light, by ventilation—in a word, by conditions which are now recognised as those which are detrimental to the bacillus; and it is not improbable that the enlarged confidence which came thus to be reposed in a vague belief as to the efficacy of "cleanliness" was not without its influence in retarding the acceptance of the doctrine which now prevails, and which seems to rest upon a sufficient basis of observation and of reasoning. From the point of view of the medical practitioner the change has been pure gain, bringing with it a fairly complete knowledge of the nature and cause of the malady, of the means of prevention, and of the methods by which, as long as cure is possible, it may be brought about. To the non-medical population, under the existing conditions of publicity, the gain, if considerable, has yet been by no means without admixture. It has opened a wide door for the exploitation of ignorance, not only by declared or obvious quacks, but also by unlearnedly sanguine members of the medical profession, whose hopes have had no inconsiderable share in the establishment of their belief in this specific or in that treatment. No Congress can save fools from the consequences of their folly; and the quack will doubtless continue to reap his harvest as he has done heretofore. The diffusion of a smattering of knowledge concerning the truth will render it only more easy to conceal, beneath this smattering, a hook baited with fraudulent pretensions. But, apart from this, and even in the case of persons of moderate intelligence, there is reason to fear that the general diffusion of knowledge of the contagiousness of tuberculosis, uncontrolled by knowledge of the conditions under which the contagion is operative, may have some tendency to the production of panic, and to the development of an entirely groundless dread of consumptives and of their surroundings. There is nothing which so quickly strips off the veneer of superficial politeness, and displays the worst side of human nature in its deformity, as the apprehension of sustaining bodily injury or of contracting disease. Not many years ago quite a number of people in London succeeded in working themselves into a state of excitement about leprosy, and they might easily, and with much more reason, do the same about consumption, until a fellow-passenger with a cough might be looked upon as a reason for vacating a railway carriage or an omnibus. With a view to such contingencies, nothing could be more opportune than the paper read by Professor Fraenkel at the Congress, and largely reported in the non-medical press, in which he called attention to the limitations of the contagiousness of tuberculosis, and to the exaggerated character of the apprehensions which have sometimes been entertained.

